# Aging in a Relativistic Biological Space-Time

**DOI:** 10.3389/fcell.2018.00055

**Published:** 2018-05-29

**Authors:** Davide Maestrini, Daniel Abler, Vikram Adhikarla, Saro Armenian, Sergio Branciamore, Nadia Carlesso, Ya-Huei Kuo, Guido Marcucci, Prativa Sahoo, Russell C. Rockne

**Affiliations:** ^1^Division of Mathematical Oncology, City of Hope, National Medical Center, Duarte, CA, United States; ^2^Department of Pediatrics, City of Hope, National Medical Center, Duarte, CA, United States; ^3^Department of Population Sciences, City of Hope, National Medical Center, Duarte, CA, United States; ^4^Department of Diabetes Complications and Metabolism, City of Hope, National Medical Center, Duarte, CA, United States; ^5^Department of Hematologic Malignancies Translational Science, City of Hope, National Medical Center, Duarte, CA, United States; ^6^City of Hope, National Medical Center, Gehr Family Center for Leukemia Research, Duarte, CA, United States

**Keywords:** special relativity, aging, biological clocks, biological space-time, manifolds, time-contraction

## Abstract

Here we present a theoretical and mathematical perspective on the process of aging. We extend the concepts of physical space and time to an abstract, mathematically-defined space, which we associate with a concept of “biological space-time” in which biological dynamics may be represented. We hypothesize that biological dynamics, represented as trajectories in biological space-time, may be used to model and study different rates of biological aging. As a consequence of this hypothesis, we show how dilation or contraction of time analogous to relativistic corrections of physical time resulting from accelerated or decelerated biological dynamics may be used to study precipitous or protracted aging. We show specific examples of how these principles may be used to model different rates of aging, with an emphasis on cancer in aging. We discuss how this theory may be tested or falsified, as well as novel concepts and implications of this theory that may improve our interpretation of biological aging.

## 1. Introduction

The connection between one's chronological age and biological age is something that we all perceive. In a sense, it is the difference between the age you “feel” and the age you *are*. Some people look “young” for their age, while some become frail earlier than others (Ness et al., 2013). Molecular “clocks” and markers of “biological age" can change throughout one's lifetime. In some cases, the rate of change of a person's biological age is *greater* than the rate of change of their chronological age. For instance, some cancers have been shown to increase biological aging, which can be described as an increase in the speed of biological clocks (Horvath, 2013).

From a mathematical perspective, biological dynamics as they relate to aging are frequently modeled as periodic or oscillating “clocks” describing, for example, circadian rhythms (Klerman and Hilaire, 2007). When studied in isolation, biological clocks can be described and predicted with periodic functions, which may speed up, slow down, or even stop and start over the course of a persons' lifetime. The more complex case of multiple integrated biological clocks can be modeled with coupled oscillators and dynamical systems theory (Shiju and Sriram, 2017). However, a fundamental assumption of these approaches is that the rate of change of these biological clocks are measured with respect to a *linear passage of time* and that the rate is independent from the biological *space* in which the biological clocks operate.

Here we investigate a mathematical model of biological space-time which includes the effects of time dilation and contraction resulting from accelerated or decelerated biological clocks, which may provide a new theoretical foundation and perspective on rates of aging. The principle assumption of our model is that a dynamic biological process may be represented as the motion of a (massless) point along a trajectory on a manifold. We then investigate the consequence of time dilation and contraction in terms of accelerating or decelerating motion of the point along the trajectory, and relate these concepts to rates of biological aging, with a particular focus on aging in cancer. In particular, we put forth the hypothesis that a biological space-time may be used to model aging from an arbitrary biological viewpoint relative to a common frame of reference. A consequence of this hypothesis is that precipitous (faster than chronological time) or protracted (slower) aging may be modeled as relativistic corrections of dilation or contraction of time along the trajectory on the manifold in which the biological aging process occurs.

To the best of our knowledge, only a few groups have proposed similar concepts. Bailly and colleagues (Bailly et al., 2011; Longo and Montévil, 2014) have proposed a mathematical definition of “biological time" as a means to model biological rhythms and periodic biological processes. However, they do not define a biological “space" nor include the possibility of the dilation or contraction of time. Systems biology pioneer Denis Noble has proposed a theory of biological relativity, which asserts that there is no “privileged level of causation" in biology (Noble, 2012). Nobel's theory contends that biological processes occur on many scales in space and in time, and that these scales are coupled to each other and should not be separated; that no single scale is responsible for the dynamics of the whole. Noble's theory implicitly couples space and time, but does not include a definition of biological space or concept of dilation or contraction of time. Consequently, our work is among the first to introduce both the mathematical interpretation and specific concept of a relativistic biological space-time to be used to study rates of biological aging.

This manuscript is structured as follows: first we describe the mathematical objects which we use to define biological space-time. After defining the meaning of relativistic dynamics in this context, we show how relativistic corrections of dilation and contraction of time can be used to model precipitous or protracted aging. We then show examples of how these principles may be used to model aspects of the aging process with a particular emphasis on aging in cancer. We discuss the implications of this theory, including criteria that may be used to test or falsify the theory and suggest novel biological quantities that may improve our interpretation of biological aging.

## 2. Biological space-time

Biology, and biological processes, are measured and observed in our conventional notion and understanding of physical space. Cells, tissues, and organisms move and change in a physical space that we can measure with length and time scales in conventional units. However, we may also consider the functional, or phenotype space in which biological processes can be represented as locations in the space. We refer to movement in a biological space as a sequence of locations in the space that form a trajectory. These general concepts have been used to characterize biological states such as hematopoietic differentiation, where 2- or 3-dimensional representations of biological space are constructed with dimension reduction techniques applied to high-dimensional single cell RNA-sequencing data (Mojtahedi et al., 2016; Nestorowa et al., 2016; Rizvi et al., 2017). The idea of the *relativity* of aging is to apply the special relativity machinery to provide a rigorously defined mathematical framework to represent biological dynamics as trajectories on manifolds moving at different acceleration rates relative to a common frame of reference. In other words, the difference in aging between two different people will be explained in terms of different dynamics in biological space-time.

### 2.1. A manifold 𝔐 and Submanifolds 𝔐_*i*_

In order to provide a conceptual picture of our mathematical framework, we imagine the space related to a biological process identified by the index *i* to be a smooth manifold $M_{i}$. A manifold is a mathematical object that is locally, but not necessarily globally, Euclidean. The canonical example of a manifold is the Earth: locally flat, globally round. If a point on Earth represents an unique locus, a point on $M_{i}$ represents a unique possible configuration or a state of a biological process. The evolution of the biological process in time, is then represented by a collection of points which form a curve, or trajectory, on the manifold $M_{i}$. If the biological process does not change in time, the trajectory degenerates to a point, with the consequent lack of motion in the biological space. We then identify the *i*-th *biological space-time* manifold by mathematically combining the time component and the spatial components of the *i*-th biological process.

Given a subset *U*_*i*_ of a topological space (i.e., a set in which at each point it is possible to associate a neighborhood) $M_{i}$, a *d*_*i*_-dimensional chart is an injective (one-to-one) function $\varphi_{i}:U_{i}\subset M_{i}\to {\mathbb{R}}^{d_{i}}$. A point ***q***_*i*_ on $M_{i}$ is identified by a set of *d*_*i*_ spatial coordinates $\left(q_{i}^{1},q_{i}^{2},\ldots q_{i}^{d_{i}}\right)$. An atlas on $M_{i}$ is the set $A_{i}=\left\{\varphi_{i,\alpha }:U_{i,\alpha }\to {\mathbb{R}}^{d_{i}}\right\}$ for some finite values of the index α, where the union *U*_*i*, 1_∪*U*_*i*, 2_∪…  is the whole space $M_{i}$. A space $M_{i}$ equipped with an atlas $A_{i}$ is a *d*_*i*_-dimensional differential manifold. For a more detailed discussion see (Tu, 2011).

A trajectory, or curve, γ_*i*_ on $M_{i}$ is a smooth map:

(1) $$\begin{array}{ll} \gamma_{i}: & I_{i}\subset {\mathbb{R}}^{+}\to M_{i}\\ \; & t\;\mapsto \gamma_{i}(t)=q_{i}(t)=(q_{i}^{1}(t),q_{i}^{2}(t),\ldots ,,q_{i}^{d_{i}}(t)). \end{array}$$

**Figure 1:**
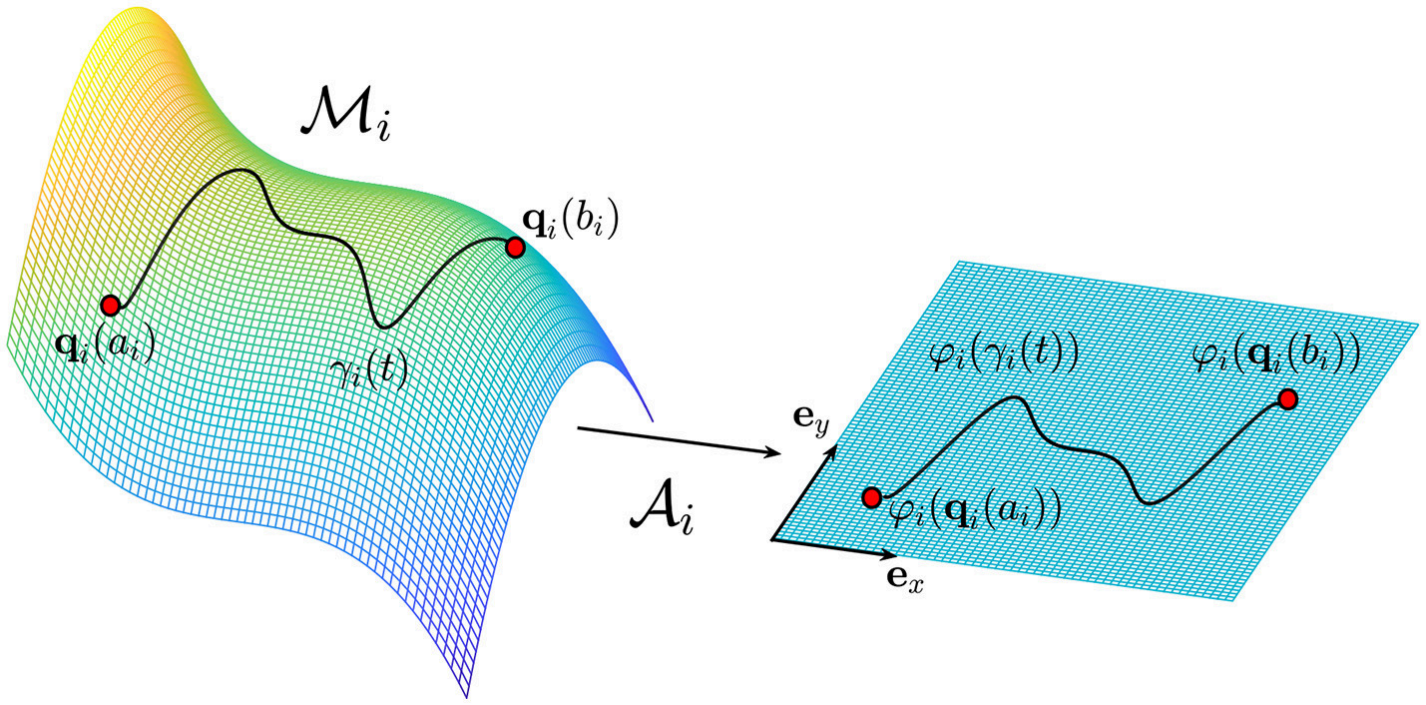
The trajectory γ_*i*_ on the manifold $M_{i}$ represents the evolution of the *i*-th biological process which starts at **q**_*i*_(*a*_*i*_) and ends at **q**_*i*_(*b*_*i*_), where *a*_*i*_, $b_{i}\in {\mathbb{R}}^{+}$ . The atlas $A_{i}$ provides the connection with the Euclidean space ℝ^2^ identified by the two unit vectors ***e***_*x*_ and ***e***_*y*_.

Here $t\in I_{i}\subset {\mathbb{R}}^{+}$ is a parameter that is interpreted as the time variable associated to the manifold $M_{i}$. The curve γ_*i*_ represents the time evolution of the *i*-th biological process starting from its beginning to its end (Figure 1). If the point ***q***_*i*_(*t*) does not change for all $t\in I_{i}\subset {\mathbb{R}}^{+}$, the trajectory degenerates to a point. We stress the fact that the variable *t* is a parameter by which is possible to parametrize the curve γ_*i*_(*t*).

Given the manifold ℝ^+^, we now define the *i*-th biological *space-time* manifold 𝔐_*i*_ as the (*d*_*i*_ + 1)-dimensional Lorentzian manifold given by the Cartesian product (the set of all ordered pairs) (see Tu, 2011):

(2) $$M_{i}={\mathbb{R}}^{+}\times M_{i}=\left\{(t,q_{i}(t))|t\in {\mathbb{R}}^{+},q_{i}(t)\in M_{i}\right\}.$$

A point $Q_{i}$ on the resulting manifold 𝔐_*i*_ is then identified by a set of *d*_*i*_ + 1 coordinates $\left(t,q_{i}^{1}\left(t\right),q_{i}^{2}\left(t\right),\ldots ,q_{i}^{d_{i}}\left(t\right)\right)$. The invariant square of an infinitesimal line element between the points $Q_{i}$ and $Q_{i}+dQ_{i}$, referred to as *space-time interval*, is then evaluated by:

(3) $$ds_{i}^{2}=\sum_{\mu ,\nu =0}^{d_{i}}g_{i,\mu \nu }dQ_{i}^{\mu }dQ_{i}^{\nu },$$

where *g*_*i*, μν_ is the metric tensor of the *i*-th biological manifold whose entries are functions of the local coordinates, $\left(t,q_{i}^{1}\left(t\right),q_{i}^{2}\left(t\right),\ldots ,q_{i}^{d_{i}}\left(t\right)\right)$. Since the metric tensor is an intrinsic property of the manifold, its provides information on the geometry of the manifold and vice-versa. It is therefore a key quantity which characterizes the biological space-time and it can be evaluated once the structure of the manifold is known or hypothesized. What we emphasize is that the notion of distance is not necessarily the usual Euclidean distance and therefore, when dealing with biological processes, distances can be measured only once the entries of the metric tensor are known.

Since a large number of biological processes take place in the body of an individual, we assume the existence of several manifolds which may be indexed $M_{i}$ and associated with individual or aggregate biological processes. We then consider all such biological processes by constructing the following manifold:

(4) $$M=M_{1}\times M_{2}\times \ldots \times M_{N},\text{ dim}(M)=\sum_{i=1}^{N}d_{i}=m.$$

A point ***q*** on M is then identified by the set of coordinates (***q***_1_, ****q****_2_, …, ***q***_*N*_) where each $q_{i}\in M_{i}$, *i* = 1, 2, …, *N* and it represents a possible configuration, or state, in which the human body can be found. Following the definition given by Equation (1), a trajectory Γ on M is a smooth map:

(5) $$\begin{array}{ll} \Gamma : & I\subset {\mathbb{R}}^{+}\to M\\ \; & t\;\mapsto \gamma (t)=q(t)=(q_{1}(t),q_{2}(t),\ldots ,q_{N}(t)), \end{array}$$

where $t\in I\subset {\mathbb{R}}^{+}$ is a parameter that is interpreted as the time variable associated to the manifold M and the curve Γ represents the entire life of a person from its birth, to its death. The connection between the curve Γ on M and the curve γ_*i*_ on $M_{i}$ will be clarified later, when the projection map will be introduced.

In analogy with Equation (2), the biological space-time 𝔐 for all biological processes occurring in a body of an individual is then defined by the *m* + 1-dimensional Lorentzian manifold given by:

(6) $$M={\mathbb{R}}^{+}\times M=\left\{(t,q(t))|t\in {\mathbb{R}}^{+},q(t)\in M\right\}.$$

We consider the Cartesian product $𝔐={\mathbb{R}}^{+}\times M$ instead of 𝔐 = 𝔐_1_ × 𝔐_2_ × ⋯ × 𝔐_*N*_ because in the latter case it is unclear how all time variables, one per each submanifold 𝔐_*i*_, will combine together and define the time on the resulting manifold 𝔐. A point Q on the manifold 𝔐 is then identified by a set of coordinates as follows:

(7) $$Q=(\overset{\in {\mathbb{R}}^{+}}{\overset{︷}{\;t\;}},\underset{\in M_{1}}{\underset{︸}{q_{1}^{1},q_{1}^{2},\ldots q_{1}^{d_{1}}}},\underset{\in M_{2}}{\underset{︸}{q_{2}^{1},q_{2}^{2},\ldots q_{2}^{d_{2}}}},\ldots ,\underset{\in M_{N}}{\underset{︸}{q_{N}^{1},q_{N}^{2},\ldots q_{N}^{d_{N}}}}).$$

**Figure 2:**
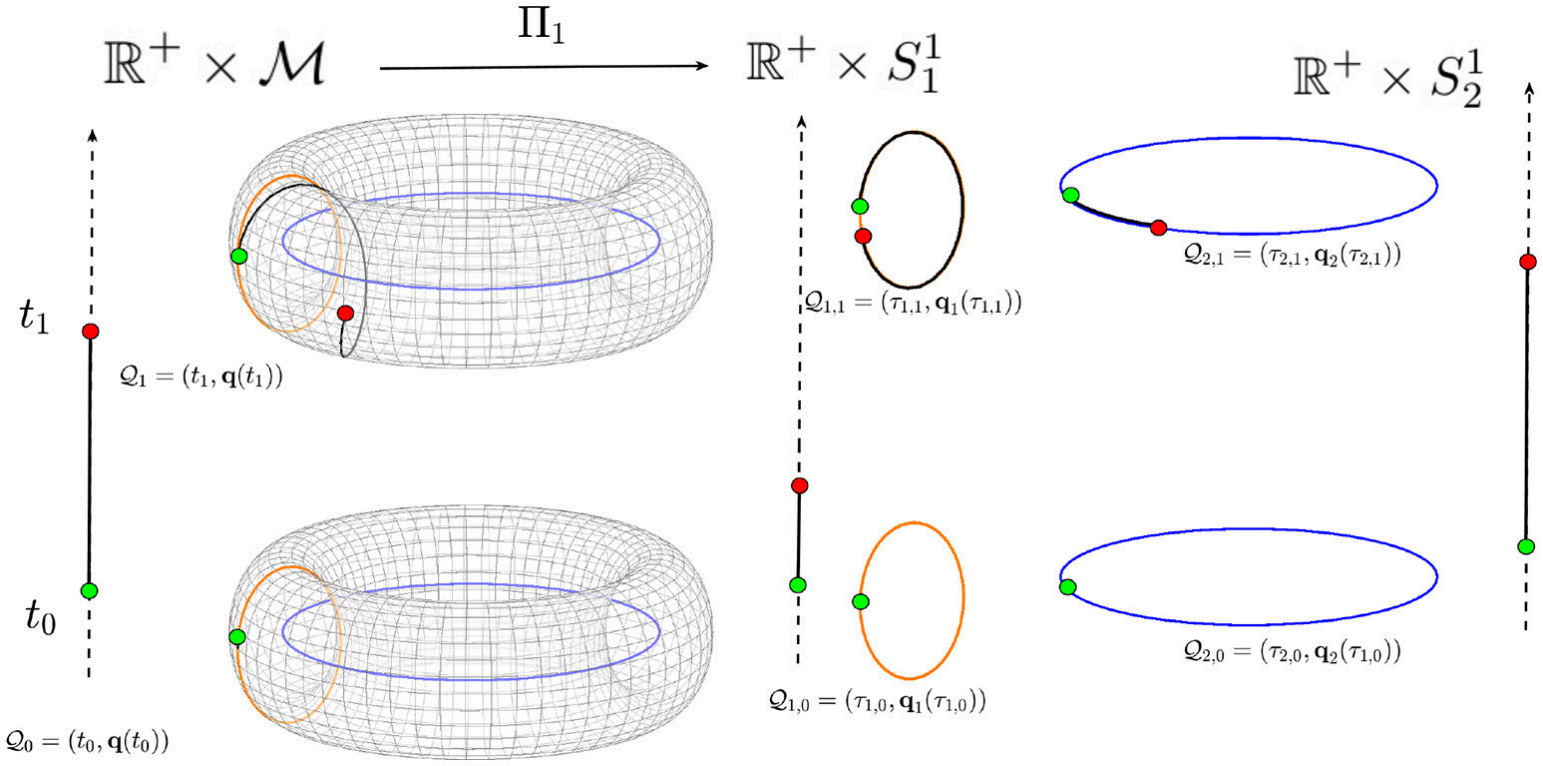
A torus is shown as an example of a possible biological space-time manifold that may be decomposed into submanifolds. The time ℝ^+^ is represented by the vertical arrows. The torus is decomposed into the two circles (submanifolds) $M_{1}=S_{1}^{1}$ and $M_{2}=S_{2}^{1}$. The point $Q_{0}$ at time *t*_0_ is mapped onto the points $Q_{1,0}$ and $Q_{2,0}$ while the point $Q_{1}$ at time *t*_1_ is mapped onto the points $Q_{1,1}$ and $Q_{2,1}$ in the two space-time submanifolds ${𝔐}_{1}={\mathbb{R}}^{+}\times S_{1}^{1}$ and ${𝔐}_{2}={\mathbb{R}}^{+}\times S_{2}^{1}$. The trajectory on the torus is then mapped onto two different trajectories on the two circles from the initial point (green circle) to the final point (red circle).

The idea for which time flows at different rates for different biological processes is now mathematically modeled by introducing a set of projection maps Π_*i*_ such that:

(8) $$\begin{array}{ll} \Pi_{i}: & {\mathbb{R}}^{+}\times M\to {\mathbb{R}}^{+}\times M_{i}\\ \; & Q\;\mapsto Q_{i}=\Pi_{i}(Q)=(\tau_{i}(t)=t_{i},\pi_{i}(q)=q_{i}), \end{array}$$

where the spatial part $\pi_{i}:M\to M_{i}$ is the canonical projection map which maps the curve Γ on M onto the curve γ_*i*_ on $M_{i}$ while the temporal part $\tau_{i}:{\mathbb{R}}^{+}\to {\mathbb{R}}^{+}$ provides the connection between the time *t* on the manifold 𝔐 and the time *t*_*i*_ on the *i*-th manifold 𝔐_*i*_ (Figure 2).

We emphasize that the relationship between *t* and *t*_*i*_ is not necessary linear. In fact, in the next section we will show that the time measured in a particular manifold depends upon the acceleration of the particle along a trajectory and it will produce a non-linear relation between *t* and *t*_*i*_.

#### 2.1.1. Example: decomposing the torus

We now provide a simple example which can be easily visualized in a three-dimensional space. We consider a manifold, the torus embedded in the three dimensional space ℝ^3^, which can be decomposed into two circles, the two submanifolds. In this particular example, this is the only possible decomposition (Tu, 2011).

The torus, embedded in ℝ^3^, is defined by the following set of equations:

(9) $$\left\{ \begin{array}{l} x(\theta ,\phi )=(R+r\cos\theta )\cos\phi \\ y(\theta ,\phi )=(R+r\cos\theta )\sin\phi \\ z(\theta ,\phi )=r\sin\phi \end{array}\right.$$

and is decomposed into the two submanifolds $S_{1}^{1}$ and $S_{2}^{1}$ given by:

(10) $$S_{1}^{1}=\left\{re^{i\theta }:\theta \in [0,2\pi )\right\},\;S_{2}^{1}=\left\{Re^{i\phi }:\phi \in [0,2\pi )\right\},$$

where, θ identifies the poloidal direction (along the orange vertical circle in Figure 2), ϕ identifies the toroidal direction (along the blue horizontal circle in Figure 2), *R* the major radius (distance from the center of the torus), and *r* < *R* is the minor radius of the torus (radius of the tube). These two circles can represent two submanifolds related to two different biological processes, and their Cartesian product produces the torus. Therefore, the two biological space-times associated to this particular example, and hence to these two biological processes, will be given ${𝔐}_{1}={\mathbb{R}}^{+}\times S_{1}^{1}$ and ${𝔐}_{2}={\mathbb{R}}^{+}\times S_{2}^{1}$. A trajectory Γ on the torus, identified by the equations:

(11) $$\left\{ \begin{array}{l} x(t)=(R+r\cos(2\pi \;nt))\cos(2\pi \;mt)\\ y(t)=(R+r\cos(2\pi \;nt))\sin(2\pi \;mt)\\ z(t)=r\sin(2\pi \;mt) \end{array}\right.$$

where *t* ∈ ℝ^+^, is then decomposed onto two trajectories:

(12) $$\begin{array}{l} s_{1}(t)=\left\{re^{2\pi \;nit}:t\in [0,1)\subset {\mathbb{R}}^{+}\right\},\\ s_{2}(t)=\left\{Re^{2\pi \;mit}:t\in [0,1)\subset {\mathbb{R}}^{+}\right\}, \end{array}$$

where *n* and *m* are the winding numbers associated with the poloidal and toroidal directions, respectively. The two submanifolds (two circles) have two different trajectories defined by Equation (12). However, when their Cartesian product is considered, the resulting manifold (the torus) has a single unique trajectory on it defined by Equation (11). In this particular example, the magnitude of the velocity and the acceleration of the projected motion on the two submanifolds are given by:

(13) $$\left\{ \begin{array}{l} v_{1}=2n\pi \;r\\ v_{2}=2m\pi \;R \end{array}\right.\text{ and }\left\{ \begin{array}{l} a_{1}=4n^{2}\pi^{2}r\\ a_{2}=4m^{2}\pi^{2}R \end{array}\right.$$

which implies *a*_2_ > *a*_1_ for the same value of *n* and *m*. The position, the velocity and the acceleration on the torus are projected onto the two subspaces in which the dynamics are identified by different values for the position, velocity and acceleration. If in Equation (11) we set *m* = 0, the trajectory reduces to a circle around the poloidal direction. In this case, Equation (13) become:

(14) $$\left\{ \begin{array}{l} v_{1}=2n\pi \;r\\ v_{2}=0 \end{array}\right.\text{ and }\left\{ \begin{array}{l} a_{1}=4n^{2}\pi^{2}r\\ a_{2}=0 \end{array}\right.$$

This example shows that there can be motion on the torus and on one submanifold, but an absence of motion on the other submanifold.

The example of the torus illustrates how trajectories on submanifolds (e.g., representations of different biological dynamics) with different accelerations may be combined and interpreted as a single trajectory on a larger manifold, and vice versa. It must be noted that in this decomposition, the time *t* in the submanifolds is a parameter along the curve and not the biological time. In fact, as we will see in the next section, because the accelerations in the two submanifolds are different we expect time to flow at different rates in each submanifold.

## 3. Dilation and contraction of time

The fact that the Maxwell's equations are invariant under the Lorentz transformation implies that any inertial observer will measure light moving at the same constant speed *c*. Moreover, the Lorentz transformations define an invariant quantity, called *proper time*, which represents a space-time interval which assumes the same value for any inertial (non-accelerating) observer. In physics, the presence of fundamental equations such Maxwell's equations, helps us in understanding the type of coordinate transformations that leave the equations unchanged and can be used to define invariant quantities. Here, we deal with a biological space-time for which the existence of fundamental equations, coordinate transformations, and corresponding invariant quantities, are unknown.

Although many investigators have posed the question of whether or not governing laws exist in biology (Ruse, 1970; Brandon, 1997; Wagner, 2017), we contend this question has yet to be conclusively answered. Rather, we believe a more fundamental question is to investigate the nature of the space-time in which any biological phenomenon occurs. In the context of Special Relativity, it has been shown by Levy-Leblond (1976) that if the space is homogeneous and isotropic, the Lorentz transformations are characterized by a parameter with the dimensions of a velocity which is an intrinsic property of the space. Moreover, the meaning of this parameter is related to the maximum velocity at which information can travel or be transmitted (Brunner et al., 2004) and this velocity turns out to be the speed of light *c*.

### 3.1. Biological invariants and information

Although information in the context of biological processes can not be easily defined (Gatenby and Frieden, 2007), it can be related to the efficiency in the conversion of energy into order (Frieden and Gatenby, 2011). In our biological context, assuming our biological space-time to be homogeneous and isotropic, there will be a parameter analogous to *c*, denoted by $\tilde{c}$, which can be related to the rate of conversion of energy into order. We stress the fact that, although we are not sure about the meaning of information in this particular context, we assume that the meaning of $\tilde{c}$ is to provide an upper limit for the information to be transmitted. This upper limit is what we believe is an invariant quantity in the biological space-time.

**Figure 3:**
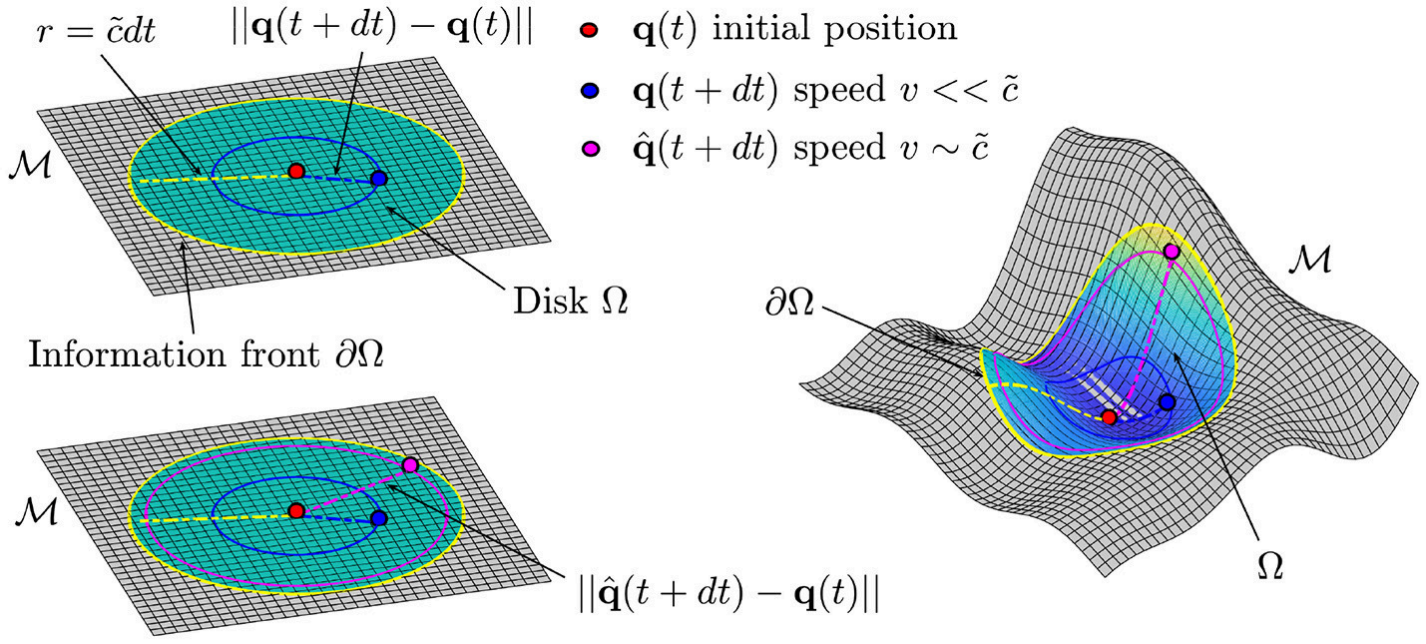
**Top left**. Representation of the information front ∂Ω propagating at speed $v=\tilde{c}$ from the center of the disk Ω on the flat plane M. The distance traveled by a point in the interval of time *dt* is given by ||***q***(*t* + *dt*) − ***q***(*t*)|| and it will be always less than $\tilde{c}dt$ (dashed yellow line), where $\tilde{c}$ is the maximum speed of information. **Bottom left**. The analogous of the relativistic corrections are needed if we want to fully characterize the dynamics of a particle which is moving at speed $v\sim \tilde{c}$ Right. In the case of a generic manifold M the information front ∂Ω will be the boundary of a region Ω.

As described in section 2.1, a point q∈M defines the state of a biological process. In what follows we assume the manifold to be a flat plane and to be constructed such that two points ***q***(*t*) and ***q***(*t* + *dt*) represent the information of the system at time *t* and *t* + *dt*, respectively (see Figure 3). The existence of a value $\tilde{c}$ defines a circular region Ω, centered at ***q***(*t*) with boundary ∂Ω given by the circumference with radius $r=\tilde{c}dt$, such that $||q\left(t\right)-q\left(t+dt\right)||<\tilde{c}dt$. In other words, the value of $\tilde{c}$ defines a front of information which propagates in time along the manifold (see Figure 3). In the case of a generic manifold M, we will have a generic region Ω bounded by a curve ∂Ω which will depend on the geometry of the manifold. In this case the distance can be evaluated once the metric tensor *g*_μν_ is known. Moreover, the existence of such a parameter defines the non-relativistic and the relativistic limits in this context. The former occurs for velocities $v<<\tilde{c}$ while the latter occurs for velocities $v\sim \tilde{c}$ (see Figure 3, top and bottom left). In analogy with the Special Relativity case, we refer to relativistic corrections, as those corrections which need to be made in order to correctly describe and characterize the dynamics of the moving particle with velocity $v\sim \tilde{c}$. We want to clarify the fact that at this stage we do not have a procedure to build a manifold for a given biological process, rather, we assume the existence of the manifold.

### 3.2. Special relativity and biological processes

Special relativity is universally recognized as a theory which describes properties of the ordinary space-time in which physical phenomena occur. In the case of a biological process, unlike the physical phenomenon, the biological space-time is not known a priori and it needs to be mathematically constructed. In this section we assume the existence of such a space and we consider the motion of two frames of reference whose coordinates are related by a set of coordinate transformations. In particular we investigate the dilation and contraction of biological time resulting from accelerated motions. This approach is used to model and to explore different rates of aging.

As shown by Levy-Leblond (1976), Lorentz transformations are the only set of transformations of coordinates in a homogeneous and isotropic space which also preserve the group structure and the causality condition. Therefore, assuming our space to be homogeneous and isotropic, we assume their validity also in a biological space-time. In this case, instead of the speed of light *c*, the Lorentz transformations will depend on the biological invariant $\tilde{c}$.

**Figure 4:**
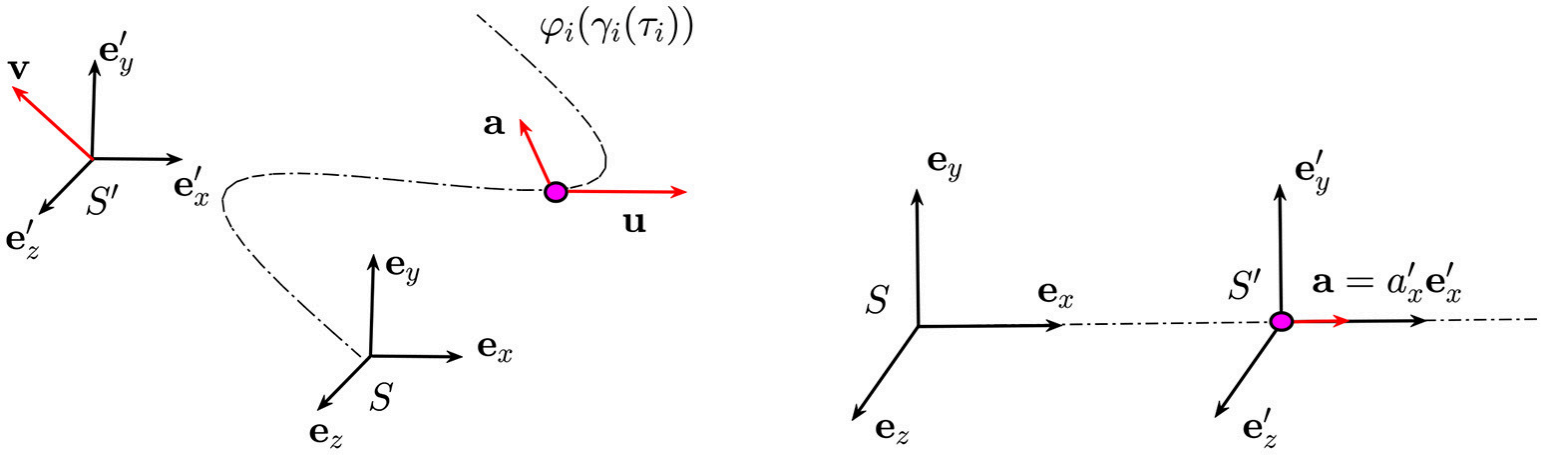
On the left) The particle (purple dot) is moving along a trajectory with velocity ***u*** and acceleration ***a***. On the right) The frame of reference *S*′ is moving at constant velocity ***v*** away from *S*: the components of the velocity and the acceleration of the particle in these two frames of references are related by Equations (A.1) and (A.2).

We consider two frames of reference *S* and *S*′ in the flat Minkowski (the space given by considering the time component and the ordinary three-dimensional space) space-time *M*^4^ = ℝ × ℝ^3^. In general, the motion of a particle is along a curvilinear trajectory, and the two frames of reference can be in a relative motion like the one represented in Figure 4 on the left. However, for simplicity, we assume a relative motion with a constant relative velocity *v* along the *x*-direction. We denote with (*t, x, y, z*) and (*t*′, *x*′, *y*′, *z*′) an event recorded by *S* and *S*′, respectively. These two events are linked by the Lorentz transformations

(15) $$\begin{array}{l} x^{\prime }=\gamma (x-vt),\;y^{\prime }=y,\;z^{\prime }=z,\;t^{\prime }=\gamma \left(t-\frac{vx}{{\tilde{c}}^{2}}\right),\\ \;\gamma =\frac{1}{\sqrt{1-v^{2}/{\tilde{c}}^{2}}}, \end{array}$$

where γ > 1 is the Lorentz factor and $\tilde{c}$ is the biological invariant previously introduced. It is straightforward to show that these transformations leave invariant the space-time interval $d\tau^{2}={\tilde{c}}^{2}dt^{2}-dx^{2}-dy^{2}-dz^{2}$ which we will call *biological proper time*. The invariance of the biological proper time will lead, also for biological systems, to the well known phenomenon of *time dilation*:

(16) $$dt^{\prime }=\gamma dt.$$

The time interval *dt*′ measured from an observer in motion is dilated by the factor γ and we say that a moving clock runs slower. It has to be remarked that this effect is reciprocal when both frames of reference are inertial (there is a relative motion at constant speed between them). In that case, no preferred frame of reference exists and each observer can argue that the other one is moving. For this reason, we do consider accelerating frames of reference in which this ambiguity is solved. We now consider the particular case in which a point is moving along a straight line oriented along the *x*-axis with a constant acceleration $a_{x}^{\prime }$ (Figure 4 on the right). We assume *S*′ to be located at the position of the particle and therefore, its velocity is the same as the velocity of the particle and it increases in time. In this particular situation, it can be shown, (see Appendix A), that the time *t*′ of the accelerated particle, and hence in the accelerated frame of reference, is given by:

(17) $$t^{\prime }=\frac{\tilde{c}}{{a^{\prime }}_{x}}\text{arcsinh}\left(\frac{{a^{\prime }}_{x}t}{\tilde{c}}\right).$$

This equation tells us how much time *t*′ elapses in the accelerated frame of reference *S*′, in terms of the time *t* elapsed in the inertial frame of reference *S*. This function represents the time component τ_*i*_(*t*) of the projection map Π_*i*_ introduced in Equation (8). In the case of an accelerating frame of reference, the phenomenon of time dilation is not reciprocal and the observer in the frame of reference *S*′ who experiences the constant acceleration cannot argue that the other observer is moving and he is still. Therefore, the time *t*′ of the accelerating frame of reference measured from the observer at rest in the frame of reference *S* appears to run slower according to Equation (17). In what follows we will apply this idea to few biological processes always assuming that the time dilation is described by Equation (17).

### 3.3. Biological age

Many markers have been used to define biological age. In our modeling framework, biological time is defined as the rate at which biological processes take place, as measured against chronological time. Therefore, our model characterizes one's biological age by the degree of dilation or contraction of time resulting from the acceleration or deceleration of biological processes that are associated with biological age.

One marker of biological age is the methlyation state of the genome, composed of varying degrees of hyper- or hypomethlyated states (Bocklandt et al., 2011; Horvath, 2013). The “epigenome" is a component of gene regulation and has been proposed to contain information relevant to the overall state of the biological system (Jenkinson et al., 2017). Linear age-related epigenetic drift has been associated with cancer incidence (Curtius et al., 2016), and statistical methods have been proposed to identify deviation from time linearity in epigenetic aging, although without a theoretical rationale to describe—or predict—the nature of the non-linearity (Snir et al., 2016).

In order to explain the process of changing methylation states with age, and the use of methylation state as a surrogate marker of biological age with our model, we first consider the trajectory on a manifold which describes the methylation process of a living person and we assume that this trajectory is mapped onto a straight line in the Minkowski space. We now consider one frame of reference *S* at rest in the origin of the trajectory and one frame of reference *S*′ that is moving at constant acceleration $a_{x}^{\prime }$ for which the time *t*′ is given by Equation (17). We then assume that the frame of reference slows down to an acceleration $a_{x}^{\prime \prime }<a_{x}^{\prime }$ for which the time is *t*″ and it is given by Equation (17). We define the rate of methylation *dM*/*dt*′ and *dM*/*dt*″ when the frame of reference moves with acceleration equal to $a_{x}^{\prime }$ and $a_{x}^{\prime \prime }$, respectively and we have:

(18) $$\frac{dM}{dt^{\prime }}=\frac{dM}{dt}\frac{dt}{dt^{\prime }},\;\frac{dM}{dt^{\prime \prime }}=\frac{dM}{dt}\frac{dt}{dt^{\prime \prime }}.$$

If $a_{x}^{\prime }>a_{x}^{\prime \prime }$ it can be shown that *dt*′ / *dt* < *dt*″ / *dt*, and therefore, we conclude that:

(19) $$\frac{dM}{dt^{\prime }}>\frac{dM}{dt^{\prime \prime }}.$$

The slowing down of the rate of demethylation is therefore interpreted as a decelerating frame of reference. We note that this model does not assume any specific functional form for the methylation trajectory. In fact, during the lifetime of a person, Equation (18) permits the acceleration or deceleration of age-related changes to the methylation state, which can be accentuated or modified in the context of cancer.

## 4. Examples of space-time dynamics in biology

In this section we connect our model to different rates of aging between individuals, and discuss examples of precipitous or protracted aging within an individual.

### 4.1. Aging

We define aging as the functional and structural decline of an organism, resulting in an increasing risk of disease, impairment and mortality over time. At the molecular and cellular level several hallmarks of aging have been proposed to define common characteristics of aging in mammals (López-Otín et al., 2013). These hallmarks include: genomic instability, telomere attrition, epigenetic alterations, loss of proteostasis, altered nutrient-sensing, mitochondrial dysfunction, cellular senescence, stem cell exhaustion and altered cell-cell communication. These processes are interdependent and influenced by cell microenvironmental cues.

Ultimately, the rate of age-related decline varies depending on how genetic variation, environmental exposure and lifestyle factors impact these mechanisms. Consequently, age, when measured chronologically, is often not a reliable indicator of the body's rate of decline or physiological breakdown. Over the years, the idea of quantifying the “biological age" based on biomarkers for cellular and systemic changes that accompany the aging process have been explored (Levine, 2013), although without a rigorous mathematical treatment of biological age. Major efforts have been made to dissect the individual contribution of each of these hallmarks and factors on aging, but the major challenge remains how to determine how their interconnectedness as a whole impacts the aging dynamics. Here we connect concepts in our mathematical model to biological and chronological aging. We note that for the sake of clarity, we will refer to aging as precipitous (faster than chronological aging) or protracted (slower) in order to clearly associate the terms “accelerated" and “decelerated" to quantities in our modeling framework.

**Figure 5:**
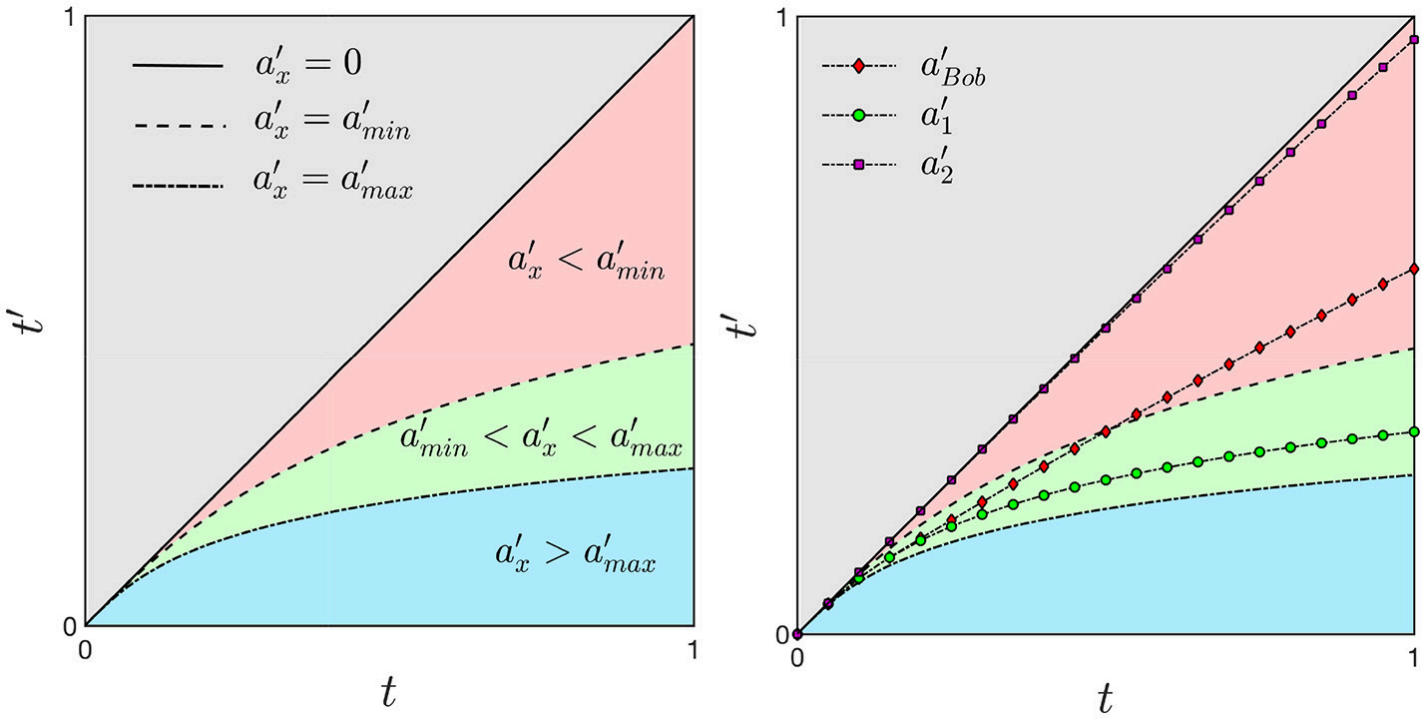
On the left) For an accelerating frame of reference a time interval Δ*t* = 1 corresponds to shorter Δ*t*′ with an consequent dilation of time. The green region represents a distribution of accelerations associated with an average range of accelerations that is used as a common reference for accelerated and decelerated processes. Smaller values of the acceleration are related to time contraction (pink region, $a_{x}^{\prime }<a_{min}^{\prime }$) while large values are associated with time dilation (blue region, $a_{x}^{\prime }>a_{max}^{\prime }$). On the right) In the mental illustration of the lifetime of Bob, a deceleration will bend Bob's trajectory (black line with red diamonds markers) out of the green region with a corresponding contraction of time.

As we derived in the previous section, an unambiguous time dilation effect requires an accelerating frame of reference. We now imagine a range of accelerations representing a distribution within a population between the values *a*_*min*_ and *a*_*max*_ (green region in Figure 5 on the left). The dashed and dotted curves corresponds to Equation (17) by using the values *a*_*min*_ and *a*_*max*_, respectively. Any acceleration within this range will correspond to a curve in the green region. An interval of time Δ*t* on the *x*-axes corresponds to a reduced interval of time Δ*t*′ on the *y*-axis and therefore, the ticking of the second clock is slowed down. The existence of a distribution, or range of values of acceleration is necessary to capture both features of time dilation, (green region in Figure 5 on the left) and time contraction, when the value of the acceleration is less than *a*_*min*_ (pink region). Moreover, values of the acceleration much larger than *a*_*max*_ can be used to define the arrest of biological time such as in a hibernating state or cryogenic freezing (blue region). The case of zero acceleration is given by Equation (16) and is not considered in this model context.

#### 4.1.1. Alice and bob

**Figure 6:**
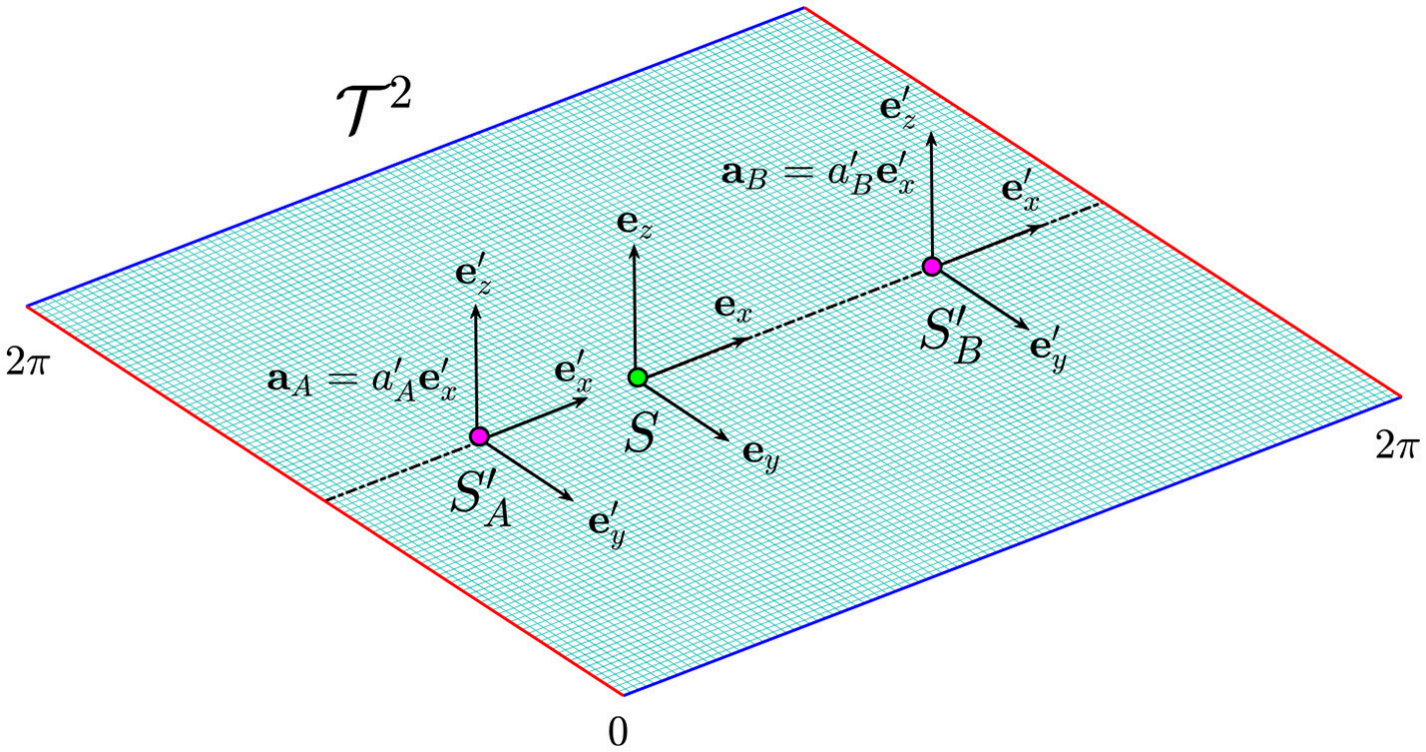
The flat torus $T^{2}$ in which the dynamics of Alice $\left(S_{A}^{\prime }\right)$ and Bob $\left(S_{B}^{\prime }\right)$ occur along the straight dotted line. The green and the purple dots represent their birth and their actual position on the manifold. The effect of time dilation is more evident for Alice whose frame of reference moves at higher acceleration.

We illustrate the contraction and dilation of time in biological space-time (i.e., on a submanifold) and how they can be related to the presence of a disease, we consider the following thought experiment involving Alice and Bob, two individuals with different rates of aging. For simplicity, we assume that their dynamics occur on a flat torus $T^{2}$ defined as the Cartesian ℝ^2^ plane under the identifications (*x, y*) ~ (*x* + 2π, *y*) ~ (*x, y* + 2π). The opposite edges *x*, *x* + 2π and *y*, *y* + 2π of the domain are identified and therefore, they must be interpreted as the same point (see red and blue edges in Figure 6). In other words, the trajectory that comes out from the right edge of the plane, will come in from the left edge and the same applies from the upper and lower part of the plane. In this case, the manifold is trivially mapped into ℝ^2^ with the identity map, and hence the dynamics on the manifold corresponds to the dynamics in the Minkowski space-time.

The point on the manifold corresponding to the birth of Alice and Bob is indicated in Figure 6 by the green circle in which we assume to place a frame of reference *S* identified by the three unit vectors (***e***_*x*_, ***e***_*y*_, ***e***_*z*_). The dynamics of Alice is represented by the motion of a purple point in which we imagine to place a frame of reference $S_{A}^{\prime }$. We also assume that the point is moving along a straight line with constant acceleration given by $a_{A}^{\prime }=a_{A}^{\prime }e_{x}^{\prime }$. The same conditions apply to Bob, but in this case the frame of reference is $S_{B}^{\prime }$ and the particle is moving with a constant acceleration $a_{B}^{\prime }=a_{B}^{\prime }e_{x}^{\prime }$ (see Figure 6).

We now can apply the results obtained in the previous section to infer that the time in $S_{A}^{\prime }$ and $S_{B}^{\prime }$ are given by Equation (17):

(20) $${t^{\prime }}_{A}=\frac{\tilde{c}}{{a^{\prime }}_{A}}\text{arcsinh}\left(\frac{{a^{\prime }}_{A}t}{\tilde{c}}\right),\;{t^{\prime }}_{B}=\frac{\tilde{c}}{{a^{\prime }}_{B}}\text{arcsinh}\left(\frac{{a^{\prime }}_{B}t}{\tilde{c}}\right).$$

Assuming the acceleration of Alice to lie within the range [*a*_*min*_, *a*_*max*_], her proper time $t_{A}^{\prime }$ lies in the green region (see Figure 5 on the right). On the other hand, Bob's frame of reference is moving with acceleration $a_{B}^{\prime }<a_{A}^{\prime }$ and its proper time $t_{B}^{\prime }>t_{A}^{\prime }$ deviates from the green region toward an older biological age in the pink region. With respect to Alice, Bob is moving with a slower acceleration and hence, his clock is running faster, resulting in a time contraction with respect to Alice's clock. If instead we assume $a_{B}^{\prime }>a_{A}^{\prime }$, Bob's proper time $t_{B}^{\prime }$ will be larger than $t_{A}^{\prime }$ and it deviates from the green toward the blue region. In this case Bob's clock will run slow with a consequent more evident effect of time dilation. Therefore, different rates of aging could be generally compared between individuals by determining their accelerations as compared to a frame of reference at rest.

We now consider only Bob and we assume his frame of reference $S_{B}^{\prime }$ to have an acceleration $a_{1}^{\prime }$ within the range $\left[a_{min}^{\prime },a_{max}^{\prime }\right]$. Suddenly, his acceleration is decreasing to the value $a_{2}^{\prime }$ and we therefore want to know what will happen to its proper time *t*′. In particular we hypothesize the acceleration to change as follows:

$${a^{\prime }}_{B}=\{\begin{array}{ll} {a^{\prime }}_{1} & t<t_{1}\\ \alpha +\beta \cos(\pi (t_{1}-t)/(t_{2}-t_{1})) & t_{1}\leq t\leq t_{2}\\ {a^{\prime }}_{2} & t>t_{2} \end{array}$$

where $\alpha =\left(a_{1}^{\prime }+a_{2}^{\prime }\right)/2$ and $\beta =\left(a_{1}^{\prime }-a_{2}^{\prime }\right)/2$. By solving Equations (A.5) and (17) we obtain the time *t*′ for the decelerating frame of reference shown in Figure 5 on the right: the line marked with green circles and the line with violet squared markers represent the trajectory corresponding to the accelerations $a_{1}^{\prime }$ and $a_{2}^{\prime }$, respectively. However, due to the deceleration, the line marked with red diamonds will not follow the line with the green circles but it will move outside the green region. The deceleration of Bob's frame of reference is reflected to a contraction of time while a larger time dilation is a consequence of a frame of reference with an increasing acceleration.

### 4.2. Cancer as inducer of precipitous aging

Major pathologies, such as cancer, diabetes, cardiovascular disorders and neurodegenerative diseases have an impact on aging. Cancer and chemotherapy in particular are known to accelerate the aging process (Alfano et al., 2017). As described above, aging involves multiple complex changes at molecular and cellular level that lead to decline in physiologic reserve capacity across virtually all organ systems (Seals et al., 2016). In cancer, accumulation of cellular damage aggravates the hallmarks of aging and accelerates aging. In cancer patients, the trajectory of decline worsens not only because of the direct physiologic insult inflicted by cancer, but also because of the injuries induced by anti-cancer therapies, such as chemotherapy or radiation, to systems that maintain physiologic reserves (Koelwyn et al., 2012; Hurria et al., 2016). In this context, there is a dose-dependent effect, whereby the more intensive the treatment of cancer or more vulnerable and frail the physiologic state, the steeper the decline in physiologic reserves (Henderson et al., 2014; Kohanski et al., 2016). Below, we use these two variables, intensity of therapy and frailty, to illustrate our model of accelerated aging.

It is not surprising that the two cancer populations most affected by precipitous aging caused by cancer are survivors of childhood cancer who are typically exposed to intensive multi-agent therapy at a young age (Henderson et al., 2014; Ness et al., 2015) and adult patients who undergo hematopoietic cell transplantation (HCT) for refractory hematologic malignancies (Arora et al., 2016). These two scenarios can be explained by our model as follows. The early onset of advanced biological age in childhood cancers corresponds to a rapid contraction of time that deviates away from the green region, similar to Bob's modified trajectory, but with an earlier onset (see Figure 5 on the right). The case of allogeneic HCT, in which case the transplanted cells come from another person, corresponds to a discontinuity in biological space-time. The recipient's trajectory on the hematopoietic manifold and/or the biological age is reset to that of the donor. If the biological ages of the donor and recipient of the HCT do not agree, the recipient will experience an abrupt and persistent change in the rate of biological aging corresponding to the discontinuity in both biological space and time.

Frailty, characterized by a cluster of measurements of physical states, is the best described measure of aging in a population, and identifies individuals who are highly vulnerable to adverse health outcomes and premature mortality. Although frailty is not a perfect corollary with biological age, it is a measure of abnormal aging at a population level. In long-term survivors of childhood cancer [median age 33 years (range 18–50 years)], the prevalence of frailty (Rockwood and Mitnitski, 2011) has been shown to be comparable to that reported among adults greater than 65 years of age (Ness et al., 2013). Comparable high rates have been reported in adult survivors of HCT (Arora et al., 2016). In fact, frail HCT recipients have a three-fold increased risk of subsequent mortality compared with the non-frail counterparts, (Arora et al., 2016) which is similar to the downstream consequences of frailty seen in the general population such as adverse health outcomes (Rockwood and Mitnitski, 2011), and early mortality (Fried et al., 2001; Hogan et al., 2003). From the lens of our model, frailty can be associated with a biological age and therefore defined as a threshold in time ($t_{f}^{\prime }$) so that the onset of frailty in the cancer population $\left(t_{c},t_{f}^{\prime }\right)$ will appear sooner relative to the general population $\left(t_{g},t_{f}^{\prime }\right)$ where *t*_*c*_ < *t*_*g*_.

#### 4.2.1. Chronic inflammation

The normal process of aging is associated with chronic low grade inflammation and with cumulative oxidative stress, independently of disease. Inflammation and oxidative stress are critical responses in host defense and injury repair and are essential for normal body functions. However, with advanced age there is a loss of sensitivity in the injury-repair cycle leading to persistent chronic inflammation, and a natural decline in the endogenous anti-oxidant capacity leading to cumulative oxidative stress (Mittal et al., 2014). These two process are interdependent, and contribute to the hallmarks of aging, influencing telomere length, mitochondrial function, epigenetics and stem cell self-renewal.

Chronic inflammation and oxidative stress are also common underlying factors of age-associated diseases. Inflammation, particularly chronic low-grade inflammation, has been found to contribute to the initiation and progression of multiple age-related pathologies such as type II Diabetes, Alzheimer's disease, cardiovascular disease and cancer (Mantovani et al., 2008). In addition, chronic diseases, and in particular cancer, elicit and promote an inflammatory tumor microenvironment that increases cancer fitness. Therefore, chronic inflammation has been suggested to underlie and accelerate biological aging (Fougère et al., 2017), in particular when associated with disease. The concept of “inflamm-aging,” inflammation-associated aging, can be used to provide a systemic perspective of biological aging in the human population (Franceschi et al., 2007).

On the level of tissue homeostasis, the best characterized example is hematopoietic aging associated with chronic inflammatory signaling in the bone marrow microenvironment. In fact, the “age" of a young hematopoietic stem cell can be “reprogrammed" when transplanted into an aged or inflammatory environment (Kovtonyuk et al., 2016), highlighting the impact of "inflamm-aging" and the plasticity of molecular clocks. Aging is associated with clonal hematopoiesis and accumulation of mutations in hematopoietic progenitors (Steensma et al., 2015), likely due to the underlying inflammation in the aged bone marrow niche. Indeed, an inflamed bone marrow can induce pre-leukemic conditions in mice similar to those occurring in elderly patients (Wang et al., 2014; Dong et al., 2016). Our model can be applied to measure how microenvironmental cues, such as inflammation, impact aging of hematopoietic cells.

### 4.3. Protracted aging

In contrast to precipitous biological aging which corresponds to the contraction of biological time, protracted aging corresponds to the dilation of biological time. This can be illustrated by prolonged periods of near zero biological activity, for instance in freezing conditions or hibernation which is part of a continuum of biological and metabolic states (van Breukelen and Martin, 2015). How can this situation be explained in a relativistic framework? We imagine a *cryogenic*-manifold mapped onto a Minkowski space. If in this space the frame of reference is moving with a high acceleration in the direction of motion then the interval of time in its frame of reference tends to zero: a frozen cell corresponds to a frame of reference moving at very *large acceleration relative to its unfrozen state* on this particular biological manifold. Figure 5 shows the limiting case of a near perfectly frozen (cryogenic) biological process (blue region), which would correspond to an extreme dilation of time, or equivalently, a very large relativistic correction.

## 5. Discussion

Here we have investigated a mathematical model of biological aging. We define a biological space-time by mathematically combining manifolds and submanifolds, and apply the principles of relativity to compare different rates of biological aging. We illustrate the concepts of precipitous and protracted aging as relativistic corrections of biological time with a mental illustration comparing the lifespans of two individuals, Bob and Alice. This analogy provides a framework to compare the rates of aging between individuals by determining their rate of acceleration as compared to a common frame of reference.

A critical component of our theory is the construction of “biological space-time,” hence a submanifold that represents a biological process. Following the work of (Levy-Leblond, 1976) we believe that the “biological space-time" should have the properties of being homogeneous and isotropic. If we also require that the transformations between frames of reference in this space preserve the group composition law and causality, the transformations are characterized by a parameter $\tilde{c}$, that has the dimensions of a velocity, which is an intrinsic property of the space (Levy-Leblond, 1976). In analogy with the case of the Lorentz transformation for which this parameter is the speed of light *c* and it represents the upper limit for the information to be transmitted (Brunner et al., 2004), we believe that $\tilde{c}$ represents the maximum speed at which information can be transmitted in the biological space-time. However, it might be possible that a space which satisfies the above constraints can not be built and hence, our theory would not apply.

On the other hand, given our ability to determine the value of $\tilde{c}$ and to represent dynamics along a trajectory on a manifold, our theory can be falsified or verified by considering the following cases. The first case occurs if the particle on the manifold moves with a constant velocity *v*. In this case, no accelerations are present and hence our theory based on accelerations does not apply. The second case takes place if the particle has an acceleration but its velocity $v<<\tilde{c}$. The particle is in a non-relativistic regime and hence, relativistic corrections are not needed. On the other hand, if accelerations are present and if $v\sim \tilde{c}$, we are in the relativistic regime and hence, relativistic corrections must be taken into account if we want to properly describe the dynamics on the manifold. We note that we construct this theory with the intention of using it to better understand biological aging. We therefore postulate that a $\tilde{c}$ may exist—or be assumed—on the same scale of observed velocities from human aging data so that we may use the theory to better study the data.

Once the submanifolds are constructed, then it is always possible to combine them considering their Cartesian product defined by Equation (4). Given our ability to construct these submanifolds, the comparison between the dynamics along different trajectories can be done if and only if they do belong to the same submanifold. So for example, if we are interested in comparing different rates of aging of two individuals with respect to the methylation status, we will need to construct the methylation status manifold, evaluate the trajectories of their methylation status in time, and analyze the dynamics on each of them. In particular, the comparison between the accelerations along both trajectories may help in understanding the difference between the rate of aging of the two individuals. In this sense, the importance is the different dynamics along each trajectory and not the fact that there are two different trajectories.

Other groups have proposed a framework for biological time to explain biological rhythms and other oscillating or biological processes that repeat periodically (Bailly et al., 2011). Our biological space-time model is more general, and is aimed towards explaining non-linear biological phenomena that are not necessarily periodic or oscillating, although these can be considered special cases of more generalized trajectories. In complement to Noble's hypothesis that there exists no privileged level of causation in biology (Noble, 2012), we suggest that the multiple scales of biology may be interpreted as trajectories on distinct manifolds that may be combined and coupled to each other. Although generalizations of relativity and space-time have been proposed, here we adapt these mathematical structures in order to interpret trajectories on manifolds to represent biological processes (O'Neill, 1983). We want to note that, as described in section 2.1.1, there can be submanifolds in which the velocity and the acceleration are identically zero. However, this does not imply an absence of motion on the other submanifolds (the other circle $S_{2}^{1}$) or on the principal manifold (the torus). In this case, it will be impossible to draw any conclusion about the possible difference between chronological and biological time for the process that takes place in the manifold in which the velocity, and hence the acceleration, is zero.

A novel concept which naturally follows from our approach is the notion of *force* in a biological context. A biological force may be a generalization of a physical force in a biological context which changes the dynamics of a particle on a manifold. We may hypothesize then, that accelerated dynamics on the manifold may be the effect of biological forces acting on the particle. For example, cancer and chemotherapy may be interpreted as forces which combine and have a resultant which may change the dynamics of the point on a manifold with a consequent impact on the rate of aging.

The shape of the manifolds which characterize biological space-time also affect our notion of distance, which requires the knowledge of the metric tensor of the manifold. Although a manifold is defined to be locally Euclidean, two biological processes or objects that are sufficiently different from each other (in space-time) may not be measured by using the conventional definition of Euclidean distance. The degree to which we must redefine our notion of a *metric* in the biological space-time may also affect our ability to interpret long-time dynamical behaviors of biological systems and whether or not relativistic corrections are required in order to properly analyze these dynamics. We therefore hypothesize that the underlying geometry of the manifold, or equivalently, the elements and structure of the metric tensor, may provide valuable insights into the biological process.

## 6. Summary

In summary, we have proposed a mathematical framework and criteria that may be used to define and construct a biological space-time. We use this as a tool to model and study different rates of biological aging based on the concept of relativistic corrections of time due to the acceleration or deceleration of biological dynamics relative to a common frame of reference. We discuss some examples of biological processes that illustrate these concepts, provide criteria that may be used to test or falsify the theory, and discuss implications and novel hypotheses that are generated by this model.

## Author contributions

RR and DM: Conception, design, and approval of final manuscript. All authors: Manuscript writing and editing.

### Conflict of interest statement

The authors declare that the research was conducted in the absence of any commercial or financial relationships that could be construed as a potential conflict of interest.

## Appendix A

By differentiating Equation (15) we obtain the transformations for the velocity

(A.1) $$\begin{array}{l} {u^{\prime }}_{x}=\frac{dx^{\prime }}{dt^{\prime }}=\frac{dx-vdt}{dt-vdx/{\tilde{c}}^{2}}=\frac{u_{x}-v}{1-vu_{x}/{\tilde{c}}^{2}},\\ {u^{\prime }}_{y}=\frac{dy^{\prime }}{dt^{\prime }}=\frac{u_{y}}{1-vu_{x}/{\tilde{c}}^{2}},\\ {u^{\prime }}_{z}=\frac{dz^{\prime }}{dt^{\prime }}=\frac{u_{z}}{1-vu_{x}/{\tilde{c}}^{2}}. \end{array}$$

where *u*_*x*_ = *dx*/*dt*, *u*_*x*_ = *dx*/*dt* and *u*_*x*_ = *dx*/*dt* are the velocities along the corresponding direction. An additional differentiation leads to the transformations of the acceleration

(A.2) $$\begin{array}{l} {a^{\prime }}_{x}=\frac{d{u^{\prime }}_{x}}{dt^{\prime }}=\frac{d{u^{\prime }}_{x}}{dt}\frac{dt}{dt^{\prime }}=\frac{a_{x}\left(1-vu_{x}/{\tilde{c}}^{2}\right)+(u_{x}-v)\left(va_{x}/{\tilde{c}}^{2}\right)}{\gamma {\left(1-vu_{x}/{\tilde{c}}^{2}\right)}^{3}}\\ \;=\frac{a_{x}(1-v^{2}/c^{2})}{\gamma {\left(1-vu_{x}/{\tilde{c}}^{2}\right)}^{3}}=\frac{a_{x}}{\gamma^{3}{\left(1-vu_{x}/{\tilde{c}}^{2}\right)}^{3}}\\ {a^{\prime }}_{y}=\frac{a_{y}}{\gamma^{2}{\left(1-vu_{x}/{\tilde{c}}^{2}\right)}^{2}}-\frac{u_{y}va_{x}/{\tilde{c}}^{2}}{\gamma {\left(1-vu_{x}/{\tilde{c}}^{2}\right)}^{3}},\\ {a^{\prime }}_{z}=\frac{a_{z}}{\gamma^{2}{\left(1-vu_{x}/{\tilde{c}}^{2}\right)}^{2}}-\frac{u_{y}va_{x}/{\tilde{c}}^{2}}{\gamma {\left(1-vu_{x}/{\tilde{c}}^{2}\right)}^{3}}, \end{array}$$

where we have evaluated *dt*′/*dt* by considering the last of Equation (15) and we used the definition of the Lorentz factor γ. The sets of Equation (A.1) and (A.2) provide the connection between the velocity and the acceleration of an object measured by an observer in *S* and another observer in *S*′. We now consider the particular case in which a point is moving along a straight line oriented along the *x*-axis with a constant acceleration $a_{x}^{\prime }$ (Figure 4 on the right). We assume *S*′ to be located at the position of the particle and therefore, its velocity is the same as the velocity of the particle and it increases in time. Hence, by setting *v* = *u*_*x*_ the *x*-component of the acceleration $a_{x}^{\prime }$ becomes

(A.3) $${a^{\prime }}_{x}=\frac{a_{x}}{\gamma^{3}{\left(1-u_{x}^{2}/{\tilde{c}}^{2}\right)}^{3}}=\frac{a_{x}{\left(1-u_{x}^{2}/{\tilde{c}}^{2}\right)}^{3/2}}{{\left(1-u_{x}^{2}/{\tilde{c}}^{2}\right)}^{3}}=\frac{a_{x}}{{\left(1-u_{x}^{2}/{\tilde{c}}^{2}\right)}^{3/2}},$$

and the acceleration in *S* is given by

(A.4) $$a_{x}={a^{\prime }}_{x}{\left(1-u_{x}^{2}/{\tilde{c}}^{2}\right)}^{3/2}.$$

By integrating the above equation with respect to time and by imposing *v*(0) = 0 we obtain the velocity

(A.5) $$u_{x}=\frac{{a^{\prime }}_{x}t}{\sqrt{1+{a^{\prime }}_{x}{}^{2}t^{2}/{\tilde{c}}^{2}}}.$$

By integrating once again with respect to time and by setting $x\left(0\right)={\tilde{c}}^{2}/a_{x}^{\prime }$ we obtain the position

(A.6) $$x(t)=\frac{{\tilde{c}}^{2}}{{a^{\prime }}_{x}}\sqrt{1+\frac{{a^{\prime }}_{x}^{2}t^{2}}{{\tilde{c}}^{2}}}.$$

The time *t*′ of the accelerated particle, and hence in the accelerated frame of reference, is given by integrating Equation (16)

(A.7) $$\begin{array}{l} t^{\prime }=\int_{0}^{t}\sqrt{1-u_{x}^{2}(s)/{\tilde{c}}^{2}}ds=\int_{0}^{t}\frac{ds}{\sqrt{1+{\left({a^{\prime }}_{x}s/\tilde{c}\right)}^{2}}}\\ \;=\frac{\tilde{c}}{{a^{\prime }}_{x}}\text{arcsinh}\left(\frac{{a^{\prime }}_{x}t}{\tilde{c}}\right). \end{array}$$
